# Effects of soybean meal fermented by *L. plantarum*, *B. subtilis* and *S. cerevisieae* on growth, immune function and intestinal morphology in weaned piglets

**DOI:** 10.1186/s12934-017-0809-3

**Published:** 2017-11-09

**Authors:** Jiajia Zhu, Mingxing Gao, Ruili Zhang, Zhuojian Sun, Chunmei Wang, Fenfang Yang, Tingting Huang, Shaoqi Qu, Li Zhao, Yuwen Li, Zhihui Hao

**Affiliations:** 1 College of Veterinary Medicine, China Agricultural University, Beijing, People’s Republic of China; 2 Agricultural Bio-pharmaceutical Laboratory, Qingdao Agricultural University, Qingdao, 266109 China

**Keywords:** Fermented soybean meal, Growth performance, Immune function, Serum parameters, Intestinal morphology, Piglets

## Abstract

**Background:**

The present study compared the effects of soybean meal fermented by three different probiotics organisms with non-fermented soybean meal on growth performance, serum parameters, immune chemistry and intestinal morphology in weaned piglets.

**Methods:**

One hundred and forty-four 35-day old crossbred (*Duroc* × *Landrace* × *Yorkshire*) piglets were randomly allocated into four different dietary treatments (n = 36 per group) containing 0, 5, 10 and 15% fermented soybean meal.

**Results:**

The piglets fed fermented soybean meal showed an increase (*p* < 0.05) in average daily weight gain and a reduction in feed consumption (*p* < 0.05).The piglets fed 10 and 15% fermented soybean meal showed the greatest growth improvement with higher levels of serum alkaline phosphatase and total serum proteins. Serum urea nitrogen in the experimental group was significantly lower than control whereas serum IgG, IgM and IgA levels were all significantly higher. Moreover, villus height in the duodenum, jejunum, and ileum was significantly higher (*p* < 0.05) and the crypt depth was significantly lower (*p* < 0.05). The levels of the autophagy factor LC3B in piglets showed a downward trend in the jejunum and ileum compared to control.

**Conclusions:**

Fermented soybean meal could significantly improve the growth, immune function and intestinal health in weaned piglets, and the best effective benefits showed in 10% FSBM group.

**Electronic supplementary material:**

The online version of this article (10.1186/s12934-017-0809-3) contains supplementary material, which is available to authorized users.

## Background

Newborn piglets have a very limited ability to digest feed and have immune systems lacking inherent disease resistance until 5 or 6-weeks of age [1]. Weaning stress is often accompanied by diarrhea, infection or even death due to lack of necessary immune function during a piglets life [2]. Antibiotics are used as feed additives for weaning piglets to reduce diarrhea and improve growth performance. However, the long-term abuse of antibiotics may encourage antibiotic resistance development [3]. For example, the use of colistin sulfate as a feed additive in pig production in China has led to the increased dissemination of the MCR-1 resistance gene in *Escherichia coli* isolated from pigs. This trend can be exacerbated by transfer to humans, resulting the failure of polymyxin therapy in human clinics [4, 5]. Therefore, it is urgent that we develop a natural and healthy agent that can improve intestinal microbial imbalances. This would lessen the severity of diarrhea and infections caused by weaning stress in piglets [6].

Probiotics are alternatives to in-feed antibiotics because they are successful in improving livestock production, efficiency and welfare [7]. Probiotic feeds have shown positive effects on piglets intestinal microbiota, increasing immunoreactivity while reducing diarrhea [8]. The administration of living microbial preparations are one part of probiotic treatments. However, another more effective pathway is microbial fermentation of feed. Microbial fermentation is a successful and effective way to remove anti-nutritional factors from feeds [9–11]. Experimentally, fermented soybeans have shown beneficial effects in the control of diarrhea in *E. coli*-challenged weaned piglets [12, 13].This treatment improved intestinal morphology and digestive enzyme activities [14] and facilitated digestibility [15] caused by the elimination of glycinin, β-conglycinin, soybean trypsin inhibitor and flatulence-producing oligosaccharides in soybean meal [16].

Autophagy is a lysosomal degradation pathway that is essential for survival, differentiation, development and homeostasis [17]. A failure in autophagy is associated with a variety of diseases including the cellular immune responses caused by pathogenic microorganisms [18]. Autophagy is also a major intracellular pathway for the degradation and recycling of long-lived proteins and entire organelles. The LC3 proteins (MAP1-LC3s) are structural proteins of autophagosomal membranes [19]. ATG genes are regulators of autophagy and LC3B and ATG8F participate in autophagosome formation [20, 21].

The ideal situation would be to select probiotics for feed that would both improve soybean meal quality and enhance resistance to weaning stress by bolstering the intestinal microbiota. The use of a combination of *Lactobacillus plantarum*, *Bacillus subtilis* and *Saccharomyces cerevisiae* to generate a fermented soybean meal (FSBM) in weaned piglets has not been previously reported and a mixed probiotics fermented SBM might show some different effects on piglets. Therefore, the objective of this study was to evaluate the effects of mixed probiotics in FSBM on the growth performance, serum parameters, immune characteristics, intestinal morphology and autophagy of intestinal tissues in weaned piglets.

## Methods

### Materials

SBM was obtained from Qingdao Bohai Agricultural Development Company; *L. plantarum B. subtilis* and *S. cerevisiae* were obtained from the Mongolian Agricultural University, Shandong Academy of Agricultural Sciences and our laboratory. Anti-MAP1LC3B antibody was purchased from Biological Engineering (Shanghai).

### FSBM preparation

*L. plantarum* was grown in de Man, Rogosa and Sharpe (MRS) medium and *B. subtilis* in Luria–Bertani medium at 37 °C. *S. cerevisiae* was grown in yeast extract-peptone-dextrose (YPD) medium at 30 °C. Fermentation was initiated by soaking SBM in distilled water to a achieve 30% moisture content. Water-soaked SBM and inoculated with a 10% mixture of equal cell numbers of *L. plantarum*, *B. subtilis* and *S. cerevisiae* to achieve 10^8^ cfu/g in SBM. SBM mixtures were anaerobically solid-state fermented at 37 °C for 48 h using a previously published protocol [22, 23]. FSBM was dried at 50 °C to a moisture content of 10%. Crude protein, KOH protein solubility of FSBM were determined by official methods of analysis [24]. Glycinin, β-conglycinin and trypsin inhibitor in FSBM were tested using a commercial kit (Dragontech Ark Biological Engineering Technology Center, Beijing, China) (Table 1).

**Table 1:** Compositions of soybean meal (SBM) and fermented SBM (FSBM)

Item	SBM	FSBM
Crude protein (%)^c^	43.03 ± 0.39^b^	48.74 ± 0.02^a^
KOH protein solubility (%)	88.89 ± 0.91^a^	76.83 ± 1.25^b^
TCA soluble protein (%)	1.21 ± 0.06^b^	12.06 ± 0.08^a^
Glycinin (mg/g)^c^	150.22 ± 0.08^a^	26.98 ± 1.33^b^
β-conglycinin (mg/g)^c^	123.20 ± 1.49^a^	36.13 ± 0.69^b^
Trypsin inhibitor (mg/g)^c^	11.16 ± 0.4^a^	0.33 ± 0.02^b^
Stachyose (%)	5.79 ± 0.087^a^	0.00^b^
Raffinose (%)	1.81 ± 0.16^a^	0.00^b^

^a, b^Means within rows with different letters differed significantly (p < 0.05)

^c^On a dry matter basis

### Animals and experimental design

A total of 144 crossbred (*Duroc* × *Landrace* × *Yorkshire*) piglets were weaned at 35 days and randomly allotted to four treatment and feed diets containing 15% SBM as control; 10% SBM and 5% FSBM as test diet 1; 5% SBM and 10% FSBM as test diet 2; and 15% FSBM as test diet 3. All diets were supplemented with minerals and vitamins to meet or exceed the requirements for piglets (Table 2) [NRC (National Academy of Sciences-National Research Council) 1998], using minor modifications of previous studies [25, 26]. Piglets were fed the diets for 35 days and were given ad libitum access to granular feed and water. Six piglets of each treatment were selected randomly to humanly sacrifice for sample collection. Piglets were weighed on days 35 and 70 after weaning, and feed intake was recorded. The average daily gain (ADG), average daily feed intake (ADFI) and feed: gain ratio (FGR) was calculated from these data.

**Table 2:** Composition of the experimental basal diet

Ingredient, as fed (%)	Control	5% FSBM	10% FSBM	15% FSBM
Corn	60	60	60	60
SBM	15	10	5	0
FSBM	0	5	10	15
Expanded soybean	7	7	7	7
Fishmeal	8	8	8	8
Calcium hydroxide	3.0	3.0	3.0	3.0
Limestone	2.0	2.0	2.0	2.0
Salt	0.35	0.35	0.35	0.35
Fat powder	3	3	3	3
Premix^a^	1.65	1.65	1.65	1.65
Total	100	100	100	100

^a^Supplied the following (mg/kg): Fe, 20; Cu, 10; Zn, 100; Se, 0.3; Ca, 60; vitamin A, 10,000 IU; vitamin E, 70 mg; vitamin D3, 1500 IU; vitamin K, 0.8 mg; vitamin B1, 2 mg; vitamin B2, 4 mg; Vitamin B6, 5 mg

### Sample collection

After treatment, blood samples were collected via the anterior vena cava and centrifuged at 3000 rpm for 10 min at 4 °C and kept at − 80 °C until use [27]. An additional 5 mL blood sample from each animal was stored at 4 °C for the determination of blood cell content. Intestinal tissues were isolated rapidly and stored in 10% formalin for morphological examination.

### Histological and morphological observations and quantitative analysis

Standard histological procedures were performed as previously described [7] [28]. Briefly, longitudinal sections of duodenum, jejunum and ileum tissues (3 cm obtained from the proximal duodenum, middle jejunum and proximal ileum) were collected and flushed using physiological saline to remove the gut contents. The tissues were fixed in 4% phosphate-buffered formaldehyde (pH 7.2) and embedded in paraffin.

Each intestinal section was treated with paraffin and cross-sectioned with a microtome and stained with hematoxylin and eosin. The villus height and crypt depth were determined according to previous methods [29]. Briefly, two cross sections (duodenum, jejunum and ileum) of each sample were prepared on a slide for morphometric analysis. Ten complete crypt-villus units were randomly selected from each sample and morphometric measurements were performed using an Olympus CKX41 microscope equipped with a digital camera (Olympus, Tokyo, Japan) and JD801 morphological analysis software.

### Serum biochemical parameters

Serum biochemical parameters of samples were evaluated for alkaline phosphatase (ALP), aspartate transaminase (AST), urea nitrogen (UN), glucose (GLU), total protein [30], albumin (ALB), globulin (GLO), calcium (Ca), phosphate (P), using an automatic biochemical analyzer (7180ISE, Japan Hitachi High-Tech, Japan).

### Determination of immune characteristics

The numbers of leukocytes, lymphocytes and lymphocyte ratios in the blood were determined using an automated blood cell analyzer (XT-2000iv, Simeon, Kang, Japan). Serum immunoglobulin A (IgA), IgM and IgG were determined with a commercial ELISA kit (Nanjing Jiancheng Bioengineering Institute, China).

### Expression of LC3B on jejunum and ileum

LC3B (microtubule-associated protein 1 light chain 3B) was detected by semi-quantitative assessment of immunohistochemistry (IHC) experiments. IHC staining was performed as previously described [21]. Our aim was to observe the expression level of LC3B in the jejunum and ileum comparing FSBM groups with control. Briefly, paraffin samples of jejunum and ileum were deparaffinized and rehydrated with a graded alcohol series and heated for antigen retrieval in sodium citrate. All slides were incubated with anti-MAP1LC3B antibody at a 1:100 dilution overnight at 4 °C. The brown granules in the cytoplasm or in nuclei were regarded as positives [31].

### Statistical analysis

All results are presented as mean ± standard deviation (SD). GraphPad 5.0 was used figures. Statistical analyses were performed using SPSS V 16.0 (SPSS Inc., Chicago, USA) and the differences between groups were compared with one-way ANOVA followed by Dunnett’s multiple comparison procedure. A *p* < 0.05 was regarded as statistically significant.

## Results

### Effect of FSBM diets on the growth performance and serum indicators in weaned piglets

**Fig. 1:**
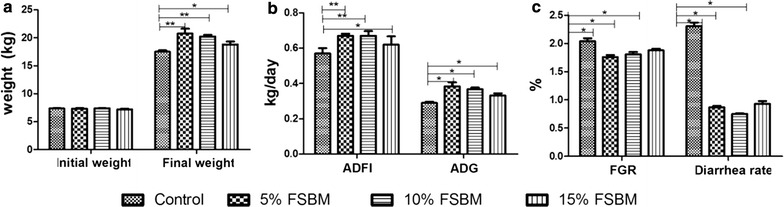
Effect of FSBM on growth performance of weaned piglets. **a** Body weight in piglets of control, 5, 10 and 15% FSBM groups (*p* < 0.05). **b** Average daily feed intake and average daily gain in piglets of control, 5, 10 and 15% FSBM groups (*p* < 0.05). **c** Different feed:gain ratio and diarrhea rate in piglets of control, 5,10 and 6 15% FSBM groups (*p* < 0.05). *p < 0.05, the difference was significant at 0.05 level. **p < 0.01, the difference was significant at 0.01 level

Compared to the SBM group, the average daily gain of piglets fed 5 and 10% FSBM diets significantly increased by 36 and 32%, respectively (Fig. 1). Piglets given 15% FSBM had about half of these increases (18%). In the FSBM groups there were proportional feed gain ratio decreases by 11.27% compared with piglets fed control diet (Fig. 1). The final weights of piglets fed dietary FSBM were also higher than controls. The 5% FSBM (p < 0.01), 10% FSBM (p < 0.01) and 15% FSBM groups (p < 0.05) all showed significant increases. Importantly, the diarrhea rate significantly decreased in FSBM groups (p < 0.05). The correlation analysis between ADG, FGR and diarrhea demonstrated that ADG was negatively correlated with FGR (p = 0.002) and diarrhea (p = 0.003) (Additional file 1: Table S1).

**Fig. 2:**
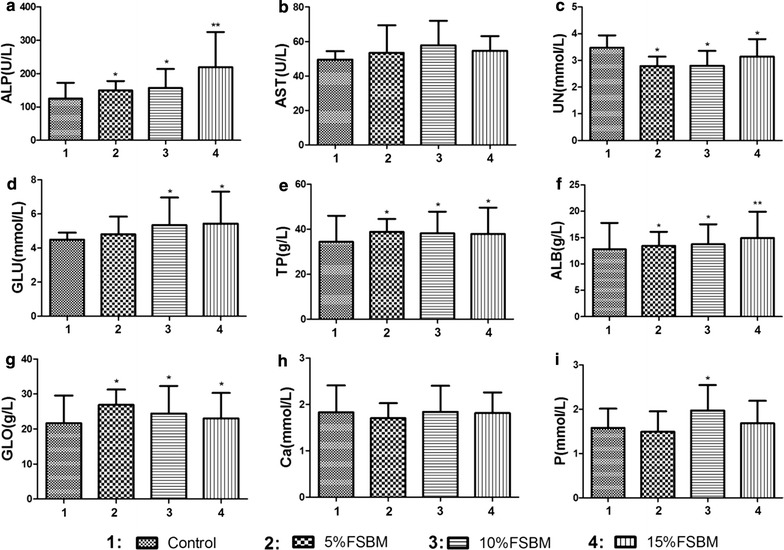
Effect of FSBM on serum parameters in weaned piglets. **a**–**i** Effect of dietary treatment on alkaline phosphatase (ALP) (**a**), aspartate transaminase (AST) (**b**), urea nitrogen (UN) (**c**), glucose (GLU) (**d**), total protein (Nakwan et al. [30]) (**e**), albumin (ALB) (**f**), globulin (GLO) (**g**), calcium (Ca) (**h**) and phosphate (P) (**i**) in weaned piglets. *p < 0.05, the difference was significant at 0.05 level. **p < 0.01, the difference was significant at 0.01 level

We found no significant differences in serum aspartate transaminase, total Ca or total p in the FSBM containing diets compared to controls. Piglets fed FSBM had higher levels of alkaline phosphatase, total protein, albumin and total globulins compared to piglets fed control diet, and the above parameters were increased by 7.84, 11.05, 9.6 and 12.90% respectively. The levels of serum glucose in piglets fed 10 and 15% FSBM increased by 17.78% although piglets fed 5% FSBM did not differ from controls. In FSBM containing diets, the levels of serum urea nitrogen were reduced by 20% and these levels were significantly lower than controls (Fig. 2).

### Immune characteristics

**Fig. 3:**
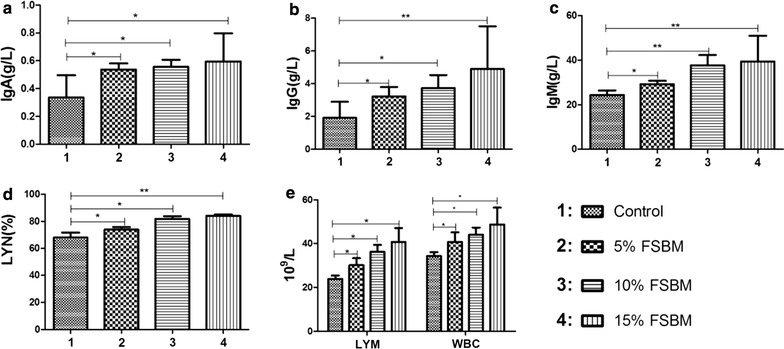
Effect of FSBM on immune characteristics in weaned piglets. **a**–**c** Effects of dietary FSBM on levels of IgA, IgG, IgM in weaning piglets. **d**–**e** Number of white blood cells (WBC) and lymphocytes, lymphocyte ratio (LYN) in serum of weaning piglets were changed in control, 5, 10 and 15% FSBM groups. *p < 0.05, the difference was significant at 0.05 level. **p < 0.01, the difference was significant at 0.01 level

Piglets fed FSBM diets had significantly higher IgA, IgG and IgM levels compared to the control diet. There were no significant differences in the IgA levels among piglets fed FSBM. However, the 15% FSBM diet showed elevated IgG levels compared to piglets fed 5 and 10% FSBM. IgM levels were higher in the latter two groups compared to piglets fed 5% FSBM (Fig. 3).

The numbers of white blood cells and lymphocytes as well as lymphocyte ratios were increased in FSBM groups. In groups fed FSBM, white blood cells were increased by 25.7, 51.5 and 70.2% in the three FSBM groups and lymphocytes increased by 18.6, 28.3 and 41.7%, respectively (Fig. 3). However, we found no significant differences in the numbers of white blood cells among piglets fed FSBM (Fig. 3). In contrast, piglets fed a 5% FSBM diet showed the lowest number of lymphocytes and piglets fed 15% FSBM had the highest (Fig. 3).

### Intestinal morphology

**Fig. 4:**
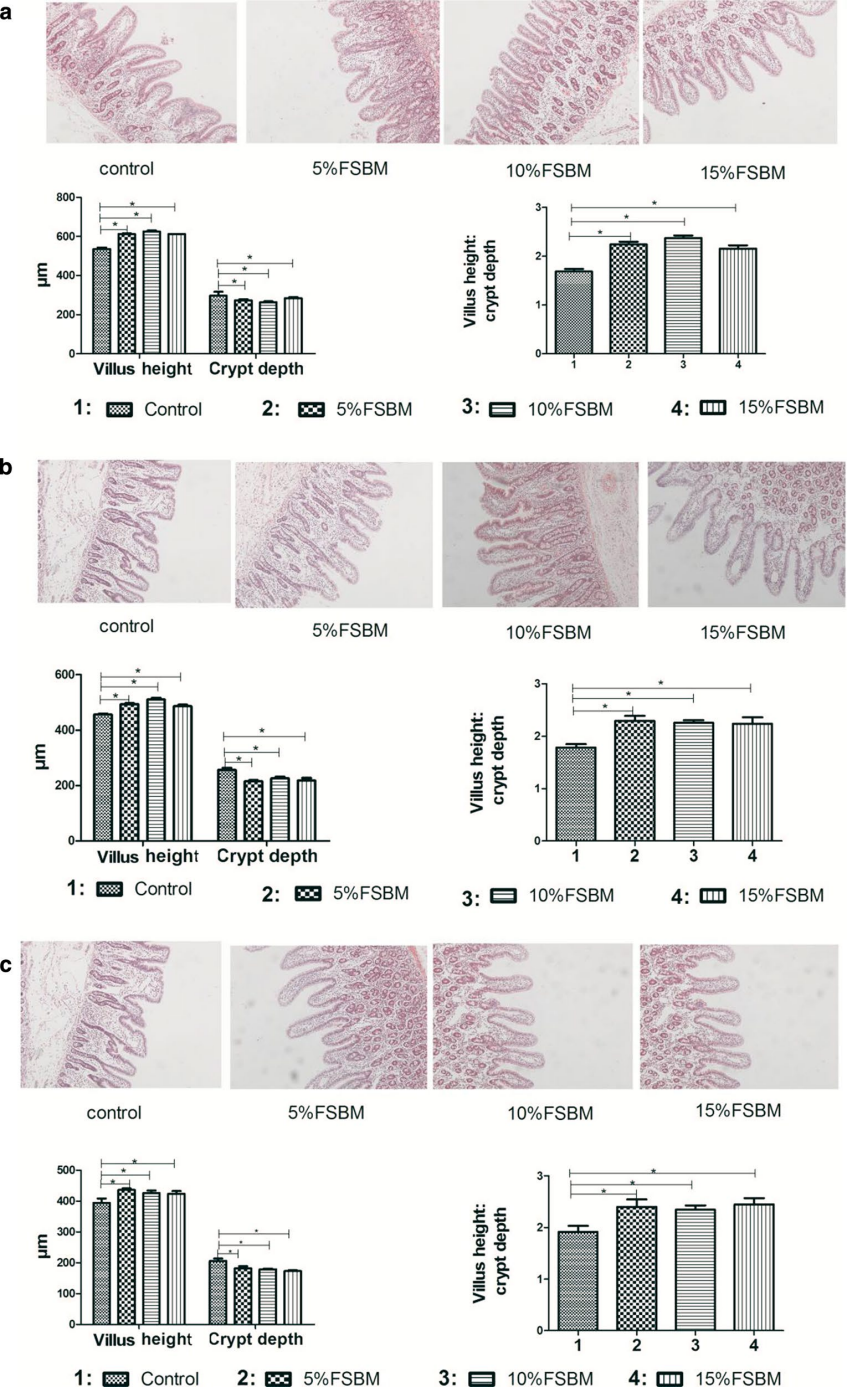
Effects of dietary FSBM on intestinal morphology of duodenum, jejunum and ileum in weaning piglets. HE 10 × 10. **a**–**c** The mucosa in the segments of **a** duodenum, **b** jejunum and **c.** *p < 0.05, the difference was significant at 0.05 level. **p < 0.01, the difference was significant at 0.01 level

Piglets fed FSBM displayed a greater villus height in the duodenum, jejunum and ileum compared to piglets fed control diet (Fig. 4). However, there were no significant differences between piglets that were fed FSBM. The crypt depth was greater in the duodenum and jejunum in control piglets. The villus/crypt ratio in all three intestinal regions were significantly greater than control values. Piglets fed FSBM had long, round, regular and tapering villi. Shorter, disordered and broader villi were apparent in piglets fed control diets (Fig. 4).

### Expression of LC3B was decreased in FSBM

**Fig. 5:**
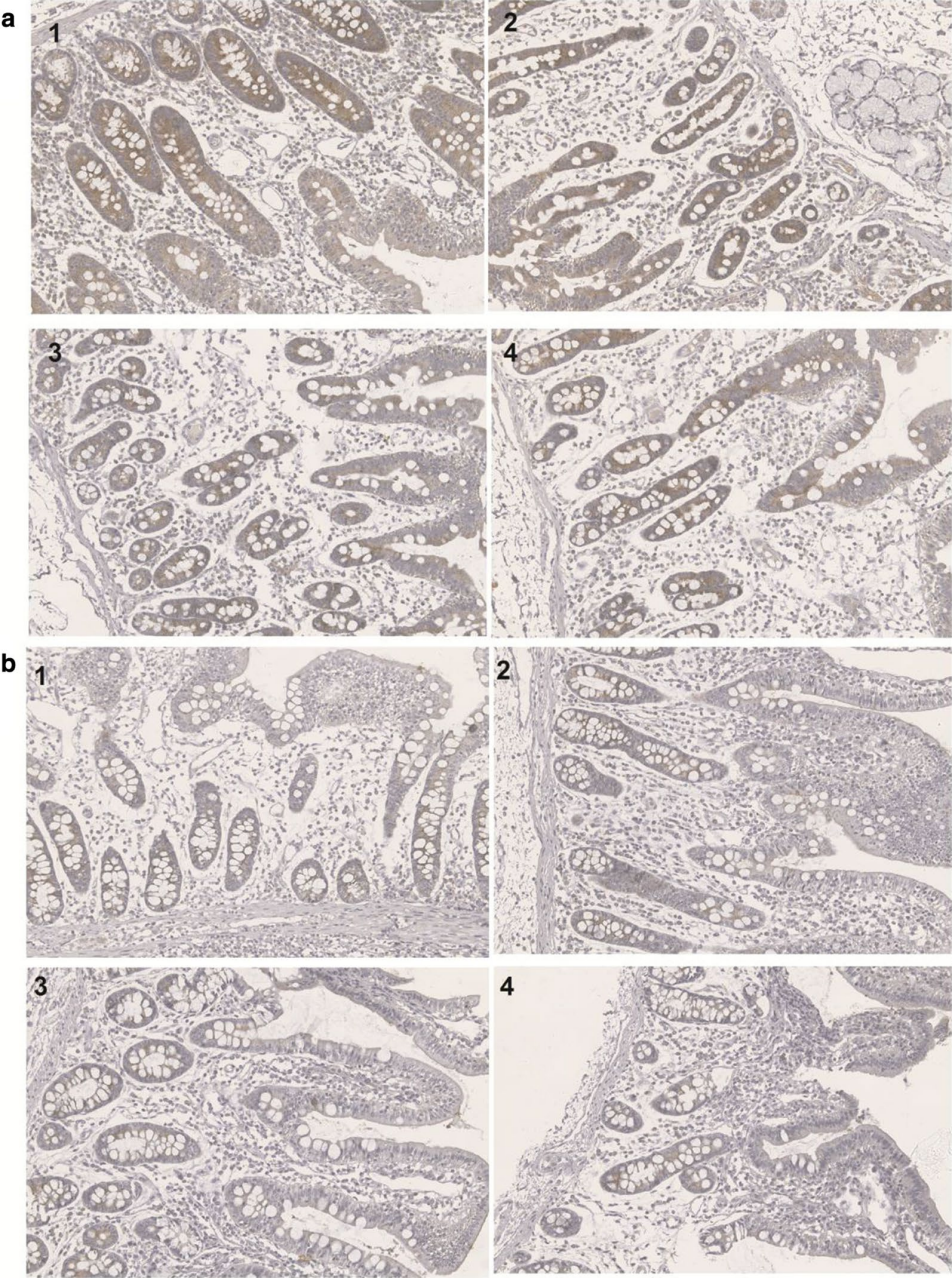
Effects of dietary FSBM on expression of LC3B in jejunum (**a**) and ileum (**b**) of weaning piglets. Visualization of LC3B expression in jejunum and ileum of piglets by immunohistochemistry (magnification ×200). 1–4 were dietary control, 5% FSBM, 10% FSBM and 15% FSBM

LC3B is a marker of autophagic activity and we found larger and denser brown areas in the photomicrographs of the controls indicating higher LC3B expression. There was a lower expression of LC3B in the ileum compared with the jejunum (compare Fig. 5a with b). There was no difference between FSBM groups in the ileum and jejunum (Fig. 5).

## Discussion

Stress is the most severe situation facing piglets during weaning and is often accompanied by harsh diarrhea [32]. Piglets may react to stressful conditions by activating physiological mechanisms to maintain homeostasis and in doing so, productivity may be compromised [33]. Currently, antibiotic resistance in piglets and the effects of stress indicate that new feed additives are needed as antibiotic replacements to treat diarrhea and the lackluster growth performance in piglets [7, 34]. In the current study, fermented soybean meal was tested as a unique technology that removes anti-nutritional factors and lessens weaning stress.

The significant increases in ADG, F:G ratio and ADFI in FSBM groups compared with controls reflected the enhanced feed utilization efficiency and improved growth performance that were similar with previous studies [35]. We found that the ADG indexes were correlated with significant improvements in F:G rates and lower rates of diarrhea. Trypsin inhibitor content and antigenic protein content was reduced to 0.33 and 20%, respectively. Stachyose and raffinose were completely absent in FSBM. The lactic acid and nutrient digestibility increased 3.75 and 7.5% respectively, and was consistent with previous reports [35, 36].

Piglets fed FSBM had increases an average daily weight gain of 3.2% and a feed gain ratio 11.3%. These values were much higher than the 1.44 and 5.56% values, respectively, previously reported [25]. However, the average daily weight gain of piglets fed 15% FSBM was lower than that of piglets fed 5 and 10% FSBM and was most likely due to excessive lactic acid in the feed. This would affect feed palatability leading to a decrease in average daily feed intake.

Diarrhea in piglets fed FSBM were decreased and the ALP, TP, ALB and GLU indexes increased, consistent with previous studies [37, 38]. ALP affects fat absorption, hydrolysis of monophosphate esters and transcellular solute transport [39]. These directly correlate with digestive and absorptive functions in the intestinal tract [40]. Interestingly, serum urea decreased in FSBM diets compared to control. This indicated that the fermentation process altered nitrogen distribution within the feed [41]. However, overall serum parameters and growth performance were consistent with each other.

In previous studies, serum immune factors including lower IgG levels and increased IgA and IgM were observed [42] [43]. We found that IgG levels increased by 93.78%. Serum IgG concentrations reflect an animal’s systemic immune status [32]. The level of white blood cells, lymphocytes, IgA and IgM were also increased by 14.32, 49.57, 69.7 and 54.8%, respectively, in our study. Lymphocyte proliferation is an important phase in the immune response of an animal and a proliferative response is antigen specific [44]. Piglet immunity is significantly reduced if β-conglycinin is not adequately inactivated during fermentation [45, 46]. Our results indicated an improvement of immune function in piglets that directly correlated with reductions of glycinin (81.89%) and β-conglycinin (70.67%) in FSBM.

Intestinal morphology reflected in villus height and crypt depth plays an essential role in nutrient absorption as well as providing a protective barrier. The facility of nutrient transport is closely related to the depth and width of villi as well as crypt depth [47]. Our study demonstrated that piglets fed FSBM had a significant increase in villi height in the duodenum, jejunum and ileum and a decrease in crypt depth compared to pigs fed SBM alone. Therefore, the ratio of villus height to crypt depth (V:C) for each of the intestinal segments increased.

We also found a negative correlation between trypsin inhibitor levels in SBM and villus height in weaning piglets [48]. SBM fermentation reduces anti-nutritional factors and therefore improves trypsin and chymotrypsin activities [49]. Our data suggests that FSBM lessens intestinal barrier injury and associated piglet diarrhea caused by weaning stress. This conclusion is consistent with other reports [2]. Accordingly, we can imagine that the expression of the autophagy related proteins might be changed in the intestinal tract [2].

LC3B expression is an indicator of an upregulation of autophagic activity. Our results indicated the piglets fed FSBM had a lower level of LC3B than that in controls both in the jejunum and ileum and there was no difference between FSBM groups. LC3B expression was higher in the jejunum than in the ileum, but the sample differences were not statistically significant. These findings indicated that the expression of LC3B in intestinal tract was changed by feeding piglets FSBM and suggested that LC3B might play a role in digestion and absorption. The decreased expression of LC3B in our experimental groups may mean less stimulation and more nutrition was supplied by FSBM to the intestinal tract. FSBM made piglets healthier than SBM but the effect on LC3B expression was subtle. This was consistent with reports that autophagy provides a survival advantage to cells with a long-time nutritional deprivation or other stresses [50]. The expression of LC3B in our results was much lower than found in vascular invasion or tumors [21]. Our piglets were all healthy without evidence of cancer. However, the differences in the results between jejunum and ileum are still unclear and need more study.

In the present study, we found an improvement in intestinal morphology that was consistent with the quality of FSBM compound probiotics. The FSBM diets showed a decreased incidence of diarrhea during weaning and improved growth performance, blood biochemical parameters, immune function and intestinal morphology of piglets. Autophagic activity of LC3B also reflected a healthy state. Overall, SBM fermentation improved digestion and nutrient utilization and a 10% FSBM supplement provided the greatest benefit.

## Additional file

**Additional file 1: Table S1.** Correlations of ADG, FGR and Diarrhea.
