# Supplementary material for: Effects of soybean meal fermented by L. plantarum, B. subtilis and S. cerevisieae on growth, immune function and intestinal morphology in weaned piglets
Source: Microb Cell Fact. 2017 Nov 9;16:191. doi: 10.1186/s12934-017-0809-3 (PMC5679485; doi:10.1186/s12934-017-0809-3)
Supplement: Supplementary file 1 — Additional file 1: Table S1. Correlations of ADG, FGR and Diarrhea. [file 12934_2017_809_MOESM1_ESM.docx]

**Additional file 1: Table S1. Correlations of ADG, FGR and Diarrhea**

| Indexes |  | ADG | FGR | Diarrhea rate |
| --- | --- | --- | --- | --- |
| ADG | Pearson Correlation | 1 | -0.792** | -0770** |
|  | Sig.(2-tailed) |  | 0.002 | 0.003 |
|  | N | 24 | 24 | 24 |
| FGR | Pearson Correlation | -0.792** | 1 | 0.828** |
|  | Sig.(2-tailed) | 0.002 |  | 0.001 |
|  | N | 15 | 15 | 15 |
| Diarrhea rate | Pearson Correlation | -0770** | 0.828** | 1 |
|  | Sig.(2-tailed) | 0.003 | 0.001 |  |
|  | N | 24 | 24 | 24 |

*p<0.05 ,the difference was significant at 0.05 level.

** p<0.01, the difference was significant at 0.01 level
